# Comparison of computer-controlled intraosseous anaesthesia and conventional anaesthesia in paediatric dentistry: a split-mouth randomised clinical trial

**DOI:** 10.1007/s40368-026-01237-4

**Published:** 2026-06-19

**Authors:** A. Dermata, S. N. Papageorgiou, A. Arhakis, N. Dabarakis, K. N. Arapostathis

**Affiliations:** 1 Paediatric Dentistry Department, Aristotle University of Thessaloniki, Thessaloniki, Greece; 2 Clinic of Orthodontics and Pediatric Dentistry, Center for Dental Medicine, University of Zurich, Zurich, Switzerland; 3 Dentoalveolar and Implant Surgery Department, Aristotle University of Thessaloniki, Thessaloniki, Greece

**Keywords:** Dental anaesthesia, Intraosseous anaesthesia, Children, Randomised clinical trial, Efficacy, Acceptance

## Abstract

**Purpose:**

To compare the efficacy, patient-reported acceptance, and preference of Computer-Controlled Intraosseous Anaesthesia (CCIA) and Conventional Local Anaesthesia (CLA) in paediatric dentistry.

**Methods:**

Healthy children aged 5–9 years who required similar treatment on both sides of either jaw were recruited from a university clinic. In a split-mouth randomised design, children received CCIA and CLA on contralateral sides one week apart, with side and sequence randomly allocated. Both procedures and data collection were performed by a single experienced paediatric dentist, and patient acceptance and preference were assessed using structured questions. Data were analysed with linear/logistic regressions adjusting for within-patient clustering (alpha = 5%).

**Results:**

64 children (mean age 7.0 years; 44% boys) were included. No significant differences in CCIA or CLA clinical efficacy were seen (*P* = 0.59). Controlled Intraosseous Anaesthesia was associated with significantly less discomfort at needle insertion (Odds Ratio [OR] = 0.32; 95% Confidence Interval (95% CI) 0.16, 0.62), during injection (OR = 0.17; 95% CI 0.08, 0.31), and overall during application (OR = 0,14; 95% CI 0.06, 0.31), as well as with lower fear levels (OR = 0.43; 95% CI 0.22, 0.84) compared to CLA (P < 0.05 in all instances). Although CCIA required longer administration, it produced shorter soft-tissue numbness and less self-inflicted trauma, and was more often associated with unpleasant taste, unusual tooth sensation, and post-injection bruising; nevertheless, CCIA was preferred by more children (78%) than CLA (22%; *P* < 0.001).

**Conclusions:**

No difference in clinical efficacy between CCIA/CLA was seen; CCIA was associated with reduced procedural pain and fear compared to CLA, resulted in shorter soft tissue numbness, less self-inflicted trauma and was preferred by most children despite longer administration time and more transient local adverse sensations.

## Introduction

Providing dental treatment with minimal pain and discomfort has always been of paramount importance in dentistry, especially amongst paediatric dentists. Dental anaesthesia is a routinely used procedure aimed at achieving effective pain control during dental treatment. Nevertheless, it is often associated with pain, anxiety, fear and discomfort in children and adolescents (Boman et al. 2008), posing a significant challenge for dental practitioners.

The most commonly used anaesthesia techniques include local infiltration anaesthesia and inferior alveolar nerve block anaesthesia. Although generally effective, these techniques are often regarded as painful due to the mechanical tissue trauma during needle insertion or to tissue distension during the administration of the anaesthetic solution (Meechan 2010). Furthermore, the conventional local anaesthesia (CLA) technique is often related to postoperative self-induced soft-tissue injury, particularly in paediatric dental patients.

Computer-controlled anaesthetic delivery systems have been developed to reduce pain by regulating both the pressure and the speed of anaesthetic solution delivery. In this context, computer-controlled intraosseous anaesthesia (CCIA) was introduced to reduce patient discomfort whilst maintaining or enhancing anaesthesia efficacy by injecting the anaesthetic solution directly into the cancellous bone adjacent to the tooth to be anaesthetised (Sixou and Barbosa-Rogier 2008). The targeted delivery results in effective pulpal anaesthesia with a reduced risk of self-induced soft-tissue trauma. Moreover, CCIA systems may demonstrate a less intimidating appearance, compared with CLA, which could reduce patients’ anxiety (Smaïl-Faugeron et al. 2019).

During computer-controlled intraosseous anaesthesia, a single injection point can provide anaesthetic coverage over an extended area, mesially, distally and lingual/palatal regions adjacent to the injection site, whereas CLA would often require multiple injection points.

Beyond anaesthetic efficacy, patient-reported acceptance and preference are important factors to consider, particularly in paediatric dental care. Although the clinical effectiveness of CCIA has been increasingly documented, the literature still lacks comprehensive evidence specifically addressing patient acceptance and preference in comparison with CLA, specifically amongst paediatric patients. Therefore, the aim of this study was to assess the efficacy, acceptance and preference of CCIA, the QuickSleeper5^®^ system compared with the CLA in paediatric dental patients.

## Materials and methods

### Trial design and participants

This study took place in the Paediatric Dentistry Department of Aristotle University of Thessaloniki between June 2023 and September 2024. The study protocol was approved by the Regional Research Ethics Committee (approval no. 94/01-10-2020) and was listed on the ISRCTN Registry (study registration number ISRCTN51792136). All participating children were patients of the postgraduate clinic of the Paediatric Dentistry Department, School of Dentistry, Aristotle University of Thessaloniki, Greece. Parents or legal guardians were thoroughly informed about the study design and objectives and then completed and signed a specific informed consent form for their child’s participation. All children were also informed about the study in an age-appropriate manner.

A randomised split-mouth controlled trial was designed. Healthy (ASA I or II) and cooperative children (Frankl behaviour rating 3 or 4), aged 5–9 years, requiring two similar dental procedures, on contralateral sides of the same jaw, as well as a third dental procedure, requiring anaesthesia, were eligible to be included in the study. Using a within-person (split-mouth) randomisation design, participants received CCIA with the QuickSleeper 5® system (Dental HiTec, Cholet, France) on one side and CLA on the contralateral side. The two treatment sessions were scheduled one week apart, and both the side of administration and the order of anaesthetic technique were randomly assigned.

All anaesthetic procedures and data collection were performed by a single experienced paediatric dentist (AD), thoroughly trained in both anaesthetic techniques.

### Intervention

Regarding CLA, local infiltration anaesthesia was used for the maxillary teeth and for the mandibular anterior teeth. Inferior alveolar nerve block anaesthesia was administered for the mandibular posterior teeth. According to the clinical protocol, a topical anaesthetic agent (xylepak^®^, Adipharm, Athens, Greece) was applied for 60 s prior to injection. CLA was administered using an aspirating syringe (Hu-Friedy Manufacturing, Co), with extra-short needles, 12 mm/30G, for infiltration anaesthesia and short needles, 21 mm/30G (Sirio Technofar^®^ Sirio Dental, Italy), for the inferior alveolar nerve block technique.

Computer-controlled intraosseous anaesthesia was administered using the QuickSleeper^®^ device. The needle was initially inserted at the interdental papilla at an angle of approximately 15°–20° relative to the mucosal surface. A small amount of anaesthetic solution was deposited, ensuring soft-tissue anaesthesia. The needle was then repositioned and advanced through the interdental septum into the cancellous bone at an angle of approximately 45° relative to the long axis of the tooth. This step was typically performed without computerised rotation due to the thin cortical plate of young children. The entire procedure was performed using the IO mode, in accordance with the manufacturer’s instructions and as described by Smaïl-Faugeron et al. (2019).

In both sessions, 4% articaine with epinephrine 1:200.000 (Artikamine^®^, Adipharm, Athens, Greece) was used as an anaesthetic agent in 1.7 ml cartridges. One-third to two-thirds of the cartridge was administered per session, depending on the extent of the treatment. The administered volume was individualised based on clinical criteria considering the type and the invasiveness of the dental procedure, in accordance with routine paediatric dental practice principles aiming to use the minimum effective dose. Consequently, no fixed volume per procedure was recorded. The same volume of anaesthetic solution was used for both sessions.

Treatment involved homologous primary teeth or first permanent molars in contralateral quadrants requiring similar treatment according to clinical and radiographic findings. The interval between the first and the second treatment session ranged from 7 to 9 days.

Randomisation of the side and sequence of anaesthetic techniques was performed using an unrestricted computer-generated list of random numbers. The operator was informed of the assigned anaesthetic method and quadrant prior to each session by an independent third party.

### Blinding

Blinding of both the operator and the children was not feasible due to the distinct nature of the two anaesthetic techniques and the delivery system. Each child received both anaesthetic methods in separate sessions using clearly distinguishable systems. The recording of each patient’s results was done in an anonymous file and each method was represented by a code. Statistical analysis was conducted by an independent investigator (SNP) who was blinded to the anaesthetic allocation until all analyses were completed.

### Covariates

The following variables were recorded: age (months), sex (male/female), type and number of dental treatments (restoration, pulpotomy, extraction, preformed metal crown [PMC]) and the method of anaesthesia used at each session (CLA or CCIA). The location of anaesthetic administration (buccal, lingual / palatal, interproximal; upper/lower jaw), and tooth type (incisor, canine, primary molar, first permanent molar) were also recorded. Anaesthetic efficacy was assessed by recording whether additional anaesthesia was required during treatment, determined by whether any pain was reported.

### Outcomes

The primary outcomes were the anaesthetic efficacy of the anaesthetic methods, patient acceptance, and patient preference. The absence of pain and the successful completion of dental treatments determined the anaesthetic methods as efficacious. If pain was reported, treatment was interrupted and supplementary anaesthesia was administered using the CLA technique.

To assess acceptance, children answered seven structured yes/no questions related to their experience with each anaesthetic technique at both treatment sessions. These included pain during needle insertion, pain during anaesthetic solution delivery, and unpleasant taste immediately after administration. The operator assessed visually if any bleeding was visible at the site of anaesthetic injection immediately after needle removal. Immediately following anaesthesia administration, children were asked whether they experienced pain during the whole anaesthetic process, felt fear, experienced pain immediately after the application, or felt soft-tissue numbness (assessed 3 min after application). Before the administration of local anaesthesia, the sensation of numbness was explained to the children using age-appropriate language as a feeling where the lip, cheek or tongue felt different, swollen or tingly, similar to the feeling experienced when a hand or foot temporarily loses normal sensation. At the end of the treatment, children were asked about the presence of numbness, pain, or any altered sensation of the treated tooth, defined as the tooth feeling higher, harder or different despite the occlusal adjustment. Furthermore, to assess numbness duration, parent/guardian and children were informed at the end of the session that they would be asked for the numbness duration in the 24 h follow-up phone call, so they were asked to document the time of numbness resolution. During the follow-up call, postoperative morbidity was assessed, including pain, use of analgesics, presence of visible mucosal lesion or bruising at the injection site, duration of the numbness, and occurrence of self-induced soft-tissue injury. Before the third treatment session, children were asked to indicate which anaesthetic method they preferred for their subsequent treatment.

### Sample size

Sample size calculation was based on a previous study (Prol Castelo et al. 2022) reporting a discomfort rate in the CLA group of 28%, an odds ratio for CCIA of 0.107, and a within-patient correlation of 0.60. Assuming these data, a total of 61 participants would be needed for alpha at 5% and beta at 20% (power of 80%). Three additional participants were added to account for potentially missing data, resulting in a sample size of 64 participants.

### Statistical analysis

Data were initially analysed descriptively, calculating means with standard deviations (SD) for normally distributed continuous variables or medians with interquartile ranges (IQR) for skewed continuous variables (after appropriate checks with the Shapiro–Wilk test) and absolute / relative frequencies for categorical variables. Differences between anaesthesia types were assessed by generalised linear models accounting for within-patient correlations with robust standard errors and expressed as either odds ratios (OR) or unstandardised regression coefficients (for categorical or continuous outcomes, respectively) with their 95% confidence intervals (CI). All analyses were run in Stata 19.5 SE (StataCorp, College Station, TX, USA) with a two-sided alpha of 5% and an open dataset (https://doi.org/10.5281/zenodo.18680402).

## Results

### Characteristics of included participants

The study group consisted of 64 participants, 36 females and 28 males, with a mean age of 7.0 ± 1.3 years. Amongst the treated teeth, 81% were primary and 19% were permanent first molars; 72% of the participants had no previous local anaesthesia experience and 52% of the treatments were performed in the mandible. Of the 128 quadrant treatments performed, 94 (73.4%) were single restorations, whilst the rest consisted of single extractions, pulpotomies, PMC placements, two restorations in two teeth in the same quadrant, a single restoration and an extraction in the same quadrant, pulpotomy combined with PMC placement, pulpotomies, and PMCs placement combined with a restoration in the same quadrant. Rubber dam isolation was used for 55 (86%) of the treatments. The values for the outcome variables are listed in detail in Table 1.

**Table 1:** Characteristics of participants

Factor	Category	Statistic
Gender *n* (%)	Male	28 (44%)
	Female	36 (56%)
Age (years) Mean (SD)		7.0 (1.3)
Jaw n (%)	Mandible	33 (52%)
	Maxilla	31 (48%)
Tooth type *n* (%)	Primary	50 (78%)
	Permanent	14 (22%)
Tooth category *n* (%)	Primary central incisor	1 (2%)
	Primary canine	1 (2%)
	1st primary molar	12 (19%)
	2nd primary molar	37 (58%)
	1st permanent molar	13 (20%)
Previous experience *n* (%)	No	46 (72%)
	Yes	18 (28%)
Treated teeth number *n* (%)	One	44 (69%)
	Two	20 (31%)
Different treatments *n* (%)	One	57 (89%)
	Two	7 (11%)
Treat: category* *n* (%)	Pulpotomy	7 (11%)
	Restoration	51 (80%)
	Extraction	7 (11%)
	PMC	7 (11%)
Restoration category *n* (%)	I	9 (18%)
	II	39 (76%)
	III	2 (4%)
	V	1 (2%)
Isolation *n* (%)	No	9 (14%)
	Yes	55 (86%)
Per cent of anaesthetic cartridge used *n* (%)	33%	23 (36%)
	40%	1 (2%)
	50%	28 (44%)
	60%	9 (14%)
	67%	3 (5%)

*PMC* preformed metal crown, *SD* standard deviation

*Overlap possible with more than one options

### Efficacy

As far as CLA is concerned, application of additional anaesthesia was needed for two participants (3%) during the dental treatment, whilst, for the CCIA, additional anaesthesia was needed in one patient (2%) (*P* = 0.59; Table 2).

**Table 2:** Descriptive statistics and univariable logistic regression analyses for differences between CLA and CCIA groups

Outcome	CLA *n* (%)	CCIA *n* (%)	OR (95% CI)	*P* value
Pain at insertion	49 (75%)	31 (49%)	0.32 (0.16, 0.62)	0.001
Pain at injection	39 (60%)	13 (21%)	0.17 (0.08, 0.39)	< 0.001
Pain felt during application	49 (75%)	19 (30%)	0.14 (0.06, 0.31)	< 0.001
Fear	23 (35%)	12 (19%)	0.43 (0.22, 0.84)	0.01
Pain immediately after application	4 (6%)	5 (8%)	1.31 (0.58, 2.99)	0.52
Bad taste	45 (69%)	56 (89%)	3.56 (1.35, 9.34)	0.01
Soft-tissue numbness 3 min after	63 (97%)	11 (17%)	0.01 (< 0.01, 0.03)	< 0.001
Pain at the end of treatment	2 (3%)	1 (2%)	0.52 (0.04, 6.14)	0.60
Altered feeling of tooth	2 (3%)	11 (18%)	6.58 (.77, 24.41)	0.005
Bleeding	6 (10%)	7 (11%)	1.21 (9.35, 4.14)	0.76
Pain at 24 h	2 (3%)	4 (6%)	2.14 (0.36, 12.75)	0.41
Painkiller used	1 (2%)	3 (5%)	3.20 (0.31, 33.13)	0.33
Bruise etc. at injection site	0 (0%)	4 (6%)	> 100 (> 100, > 100)*	< 0.001
Soft-tissue trauma	6 (9%)	0 (0%)	< 0.01 (< 0.01, < 0.01)*	< 0.001
Extra anaesthesia needed	2 (3%)	1 (2%)	0.51 (0.04, 6.02)	0.59

*CCIA* computer-controlled intraosseous anaesthesia, *CI* confidence interval, *CLA* conventional local anaesthesia, *OR* odds ratio

*Relative risk used instead, as odds ratio could not be used

### Acceptance

49 participants (75%) reported pain at the moment of needle insertion for CLA and 31 (49%) for CCIA (OR = 0.32; 95% CI 0.16, 0.6; *P* = 0.001). During the anaesthetic injection, 39 participants (60%) reported pain for CLA and 13 (21%) during CCIA (OR = 0.17; 95% CI 0.08, 0.31; *P* < 0.001). Bad taste was reported by 45 (69%) participants for CLA and by 56 (89%) for CCIA (OR = 3.56; 95% CI 1.35, 9.34; *P* = 0.01). Bleeding during the application occurred in 6 participants (10%) for CLA and in 7 participants (11%) for CCIA (*P* = 0.75).

Mean duration of the total anaesthetic procedure was 2 min and 20 s for CLA and 3 min for the CCIA (*P* < 0.001).

Immediately after the application, 49 participants (75%) reported discomfort during the CLA and 19 (30%) during CCIA (OR = 0.14; 95% CI 0.06, 0.31; *P* < 0.001). 23 participants (35%) reported fear during CLA and 12 (19%) during CCIA (OR = 0.43; 95% CI 0.22, 0.84; *P* = 0.01). Four participants (6%) expressed current pain immediately after the application of CLA and 5 (8%) expressed current discomfort immediately after CCIA (*P* = 0.52). Three minutes after the application of the CLA method, 63 participants (97%) felt soft-tissue numbness, and 11 (17%) felt soft-tissue numbness 3 min after CCIA (OR = 0.01; 95% CI 0.01, 0.03; *P* < 0.001).

Two participants (3%) expressed pain immediately after the dental treatment under CLA, whilst 1 patient (2%) under CCIA did (*P* = 0.60). Two participants (3%) reported altered tooth sensation, defined as a tooth that felt higher, harder or different, after dental treatment with the CLA method and 11 (18%) with CCIA did (*P* = 0.005).

None of the participants reported a visible mucosal lesion or bruise in the injection site 24 h after CLA whilst 4 (6%) reported a bruise in the injection site 24 h after the CCIA (*P* < 0.001). 24 hours after application, 6 participants (9%) reported self-induced soft-tissue trauma for the CLA method and none for the CCIA (*P* < 0.001). One participant (2%) reported use of a painkiller in the last 24 h after CLA and 3 (5%) after CCIA (Table 2).

The mean numbness duration was 2 h for CLA. No postoperative numbness was reported for CCIA (*P* < 0.001; Table 3).

**Table 3:** Descriptive statistics and univariable generalised linear regression analyses for differences between groups

Outcome	CLA Median (IQR)	CCIA Median (IQR)	Difference (95% CI)	P
Anaesthesia procedure duration (seconds)	140.0 (120.0, 180.0)	180.0 (170.0, 210.0)	39.52 (28.8, 50.3)	< 0.001
Soft-tissue numbness duration (hours)	2.0 (1.4, 2.5)	0 (0, 0)	−1.8 (−2.0, −1.6)	< 0.001

*CCIA* computer-controlled intraosseous anaesthesia, *CI* confidence interval, *CLA* conventional local anaesthesia, *IQR* interquartile range

### Preference

14 participants (22%) preferred the CLA and 50 (78%) reported preference for the CCIA (*P* < 0.001). Children who underwent extraction tended to prefer the CCIA over the CLA (relative risk = 1.39; 95% CI 1.20, 1.61; *P* < 0.001). Assessment of other factors potentially associated with the participants choosing CCIA over CLA can be seen in Table 4.

**Table 4:** Assessment of potential confounders on the patient’s preferred anaesthesia

Covariate	Category	OR (95% CI)	*P*
Age	Per year	1.13 (0.71, 1.80)	0.61
Sex	Girl	Reference	
	Boy	2.31 (0.63, 8.43)	0.21
Jaw	Mandible	Reference	
	Maxilla	0.44 (0.13, 1.50)	0.19
Dentition	Primary	Reference	
	Permanent	0.63 (0.16, 2.44)	0.50
Previous experience	No	Reference	
	Yes	1.57 (0.38, 6.53)	0.53
Teeth treated	One	Reference	
	More	0.52 (0.15, 1.78)	0.30
Treatment types	One	Reference	
	More	1.77 (0.19, 16.37)	0.61
Treatment: pulpotomy	No	Reference	
	Yes	1.77 (0.19, 16.37)	0.61
Treatment: restoration	No	Reference	
	Yes	0.24 (0.03, 2.10)	0.20
Treatment: extraction	No	Reference	
	Yes	1.39 (1.20, 1.61)*	0.001
Treatment: PMC	No	Reference	
	Yes	1.77 (0.19, 16.37)	0.61
Isolation	No	Reference	
	Yes	2.00 (0.43, 9.40)	0.38
First method	CLA	Reference	
	CCIA	0.72 (0.22, 2.40)	0.60

*CCIA* computer-controlled intraosseous anaesthesia, *CI* confidence interval, *CLA* conventional local anaesthesia, *NT* could not be tested, *OR* odds ratio, *PMC* preformed metal crown

*Relative risk used instead, as odds ratio could not be used

## Discussion

Both anaesthetic methods demonstrated efficacy with regards to the completion of dental treatment. Additional anaesthesia was required in two participants (3%) in the CLA group and in one participant (2%) in the CCIA group, without a statistically significant difference. The study of Smaïl-Faugeron et al. (2019) also claimed similar efficacy of CLA and CCIA. Several studies performed in children and adults showed that CCIA can be used as an alternative to CLA as the anaesthetic solution is administered in the cancellous bone in close proximity to the root of the tooth requiring anaesthesia (Sixou and Barbosa-Rogier 2008; Sixou et al. 2009; Collier 2018; Smaïl-Faugeron et al. 2019). This technique has demonstrated adequate efficacy even in teeth with irreversible pulpitis or hypomineralisation (Cabasse et al. 2015; Dixit and Joshi 2018). It has also been suggested that CCIA demonstrates an anaesthetic result that is more certain and predictable (Dixit and Joshi 2018; Attia et al. 2022). Conversely, some studies have reported that the anaesthetic duration of CCIA may be insufficient for longer procedures (Özer et al. 2012; Sovatdy et al. 2018). In the present study, CCIA duration was sufficient for the successful completion of dental treatments and did not differ significantly from that of CLA, a result similar to that of other studies (Beneito-Brotons et al. 2012; Prol Castelo et al. 2022).

The importance of the efficacy of the anaesthetic methods is indisputable. Nevertheless, the patient’s acceptance is also critical, particularly in paediatric dentistry. The degree of acceptance of the anaesthetic methods can be assessed by evaluating every single stage of the anaesthetic process, before, during and after administration, as perceived and tolerated by the patient. To determine the acceptance, factors, such as pain, discomfort, fear, feeling of numbness, bad taste, and postoperative morbidity, are to be considered. Although local anaesthesia aims to control pain, it is commonly associated with anxiety, discomfort and pain, especially in children (Arapostathis et al. 2010).

Any reduction in pain during dental anaesthesia should be considered in favour of the patient (Versloot et al. 2008; Arapostathis et al. 2010; Smaïl-Faugeron et al. 2019). When applying local anaesthesia, pain is attributed to the mechanical trauma of the tissues due to the needle insertion, as well as the tension of the tissues during the injection of the anaesthetic solution. The Quicksleeper® system is suggested to offer support for the needle insertion and also to assist the injection procedure, as the pressure applied is stable and controlled by the device (Sixou and Barbosa-Rogier 2008; Smaïl-Faugeron et al. 2019). In the present study, significantly fewer participants reported pain during needle insertion and solution delivery with CCIA compared with CLA. Overall discomfort during anaesthesia was also reported more frequently with CLA than CCIA. This is in accordance with the studies of Smaïl-Faugeron et al. (2019) and Prol Castelo et al. (2022) who reported reduced pain associated with CCIA in paediatric participants. Similarly, the preliminary study of Beneito-Brotons et al. (2012) demonstrated less discomfort associated with the application of the CCIA versus the conventional alveolar nerve block anaesthesia in adult participants.

The procedure of local anaesthesia, although mostly efficacious is, as aforementioned, linked with anxiety and may result in a negative experience, fear of future anaesthetic procedures, and reluctance to seek dental care (Arapostathis et al. 2010; Carrillo-Díaz et al. 2012). The physical appearance of the plastic CCIA device has been described as less intimidating than that of conventional syringes, therefore, could be associated with less anxiety, particularly in paediatric patients. In the present study, 35% of paediatric dental patients reported fear during CLA administration compared with 19% during CCIA administration (*P* = 0.01). Other studies have found that CCIA units are associated with more acceptance and less anxiety (Kuşcu and Akyuz 2006; França et al. 2022; Ludovichetti et al. 2022).

Regarding the anaesthesia’s application time, CLA was administered more rapidly than CCIA (mean duration 2 min 20 s versus 3 min). The duration of local anaesthesia application for CLA was calculated considering all procedural steps, such as buccal, intrapapillary, and palatal injections, for the maxilla and the inferior alveolar nerve block and buccal injection for the mandible. In contrast, CCIA administration time included both intrapapillary and intraosseous delivery. The anaesthesia application time is in accordance with the study by Pol et al. (2022), which also suggested that CLA required less time to be administered when compared to CCIA.

An unpleasant taste was reported more frequently during CCIA administration (89%) than during CLA (69%). Leakage of anaesthetic solution during CCIA has also been supported in other studies (Nusstein et al. 2010; Özer et al. 2012). Eleven participants (18%) felt their anaesthetised tooth to be “high”, “hard” or “strange” immediately after the treatment with the use of CCIA, whereas 2 participants (3%) reported a strange feeling in the tooth after CLA.

Soft-tissue numbness affecting the lips, cheeks or tongue after CLA is commonly associated with discomfort and impaired oral function. Furthermore, with regard to paediatric dental patients, this altered sensation cannot clearly be differentiated from pain and sometimes provokes self-induced soft-tissue trauma. (Ram et al. 2012). In the present study, we perceived as numbness the patients’ reports of regional tingling, swelling, strange sensation or numbness of the lip, cheek and tongue. Almost all participants reported numbness three minutes after CLA administration, compared with a significantly smaller proportion following CCIA. Parents/guardians were informed that they had to note the time of numbness resolution. Mean numbness duration as reported from the parents 24 h after the dental treatment was 2 h for the CLA. No numbness was reported for the CCIA (duration noted as zero). Reduced numbness duration has also been demonstrated in other studies (Smaïl-Faugeron et al. 2019; Pol et al. 2022; Prol Castelo et al. 2022). However, some studies stated that the limited anaesthetic duration could, in some cases, be suggested as a limiting factor for the CCIA as it could possibly lead to postoperative pain shortly after an invasive dental procedure before the administration of analgesics (Peñarrocha-Oltra et al. 2012; Pol et al. 2022).

Self-induced soft-tissue trauma was reported by six participants following CLA and by none following CCIA (*P* < 0.001), a result that is in compliance with the studies of Sixou and Barbosa-Rogier (2008) and Prol Castelo et al. (2022). There was no statistically significant difference in the reported postoperative pain. Six per cent of the participants reported bruise at the injection site for CCIA and none for the CLA 24 h after the application (*P* < 0.001).

In the present study, when participants were asked which method they would prefer for a third dental treatment, 50 (78%) expressed their preference for the CCIA in comparison with 14 (22%) who preferred the CLA, and the difference was statistically significant (*P* < 0.001). The confounder factors assessment only indicated the extraction as a possible confounder towards the preference of CCIA. In the study of Beneito-Brotons et al. (2012) in adult patients, 69.7% of the participants preferred the CCIA versus 23.3% of the participants who reported a preference for the CLA. In the study of Prol Castelo et al. (2022), most of the paediatric dental patients also reported a preference for the CCIA (76%) in comparison with the CLA. Results are in accordance with the present study.

Despite the strengths of the present randomised split-mouth design, certain limitations should be acknowledged. First, all anaesthetic procedures were performed by a single experienced paediatric dentist. Although this approach ensured consistency in technique and minimised inter-operator variability, it may limit the generalisability of the findings to operators with different levels of experience. Second, blinding of both the operator and the participants was not feasible due to the distinct characteristics of the anaesthetic delivery systems, which may have introduced a degree of expectation bias, particularly in patient-reported outcomes, such as acceptance and preference. However, this limitation is inherent in comparative studies of anaesthetic techniques with visibly different devices. In addition, pain and acceptance were assessed using binary yes/no responses rather than validated pain scales. This approach was selected to ensure comprehension and reliability of responses in young children aged 5–9 years, but it may have reduced sensitivity in detecting subtle differences in pain perception. Furthermore, generalisation of the results could be jeopardised by the age range and the level of the patient’s cooperation. Similar studies in paediatric patients younger than 5 years old are scarce (Klein et al. 2005) as well as clinical trials including patients with limited cooperation (Frankl 1 or Frankl 2) (Prol Castelo et al. 2022).

## Conclusion

No significant difference in clinical efficacy was seen between CCIA and CLA in paediatric dentistry. Compared to CLA, CCIA significantly reduced discomfort and fear during administration and resulted in shorter soft-tissue numbness and less self-inflicted trauma, despite longer administration time and more frequent transient adverse sensations. Overall, more children preferred CCIA over CLA.

## Data Availability

The full dataset of this study is openly available (https://doi.org/10.5281/zenodo.18680402).
